# Visualizing cellular interactions: intravital imaging in tumor microenvironment

**DOI:** 10.3389/fimmu.2025.1630658

**Published:** 2025-09-09

**Authors:** Shichao Li, Limei Liu, Juanjuan Shan, Cheng Qian

**Affiliations:** ^1^ Chongqing Key Laboratory of Translational Research for Cancer Metastasis and Individualized Treatment, Chongqing University Cancer Hospital, Chongqing, China; ^2^ Department of Breast and Thyroid Surgery, Southwest Hospital of Army Medical University, Chongqing, China

**Keywords:** intravital imaging, tumor microenvironment, cellular interaction, tumor, *in vivo* imaging

## Abstract

The tumor milieu is a dynamic ecosystem where immune cells, stromal cells, and tumor cells interact to influence tumor progression and anti-tumor immunity. Traditional experimental methods, limited to static *in vitro* or ex vivo analyses at specific time points, cannot fully capture the complexity and dynamic evolution of the tumor microenvironment (TME) in living organisms. Intravital microscopy (IVM), powered by advanced imaging technologies, precise labeling strategies, and optimized experimental approaches, enables real-time visualization of biological structures and cellular interactions within living animals. This review synthesizes findings from IVM-based research, focusing on the dynamic and transient interactions between tumor cells and other cell types, such as normal epithelial cells, immune cells, and stromal cells. It explores the nature of these interactions, their impact on tumor progression, and the outcomes of therapeutic interventions.Overall, we aim to provide a comprehensive resource that highlights the role of IVM in uncovering the dynamic cellular interplay within the TME and its implications for advancing tumor biological research and improving cancer therapies.

## 1 Background

Tumors represent complex ecosystems comprising tumor cell parenchyma and microenvironment constituted by epithelial cells, immune cells, endothelial cells, fibroblasts and extracellular matrix (ECM) (1, 2). A growing body of evidence indicates that the disease course depends on the interaction between tumor cells and the TME. The TME is not static and can change over time (3, 4). While long-range cell-cell interactions have been described, e.g. through extravesicular vesicles, cellular interactions primarily occur through direct physical contact and paracrine signaling via soluble factors (5). Contact-dependent interactions are mediated by adhesion molecules, including integrins, selectins, cadherins, and members of the immunoglobulin superfamily, as well as through gap junctions and tunneling nanotubes (6). Paracrine signaling, driven by intrinsic tumor characteristics, cellular stress responses, and the release of cytokines, chemokines, growth factors, and proteolytic enzymes from various cell types, facilitates communication within the tumor milieu (7).

The initiation, progression, and metastasis of tumors are intricately linked to the complex interactions between tumor cells and various components of the TME (8). Cells within the TME and tumor cells can mutually influence each other, ultimately promoting or inhibiting tumor initiation, progression, and treatment outcomes (9–11). With the advancement of multidimensional flow cytometry and single-cell sequencing in recent years, our understanding of the molecular mechanisms underlying tumor-TME interactions has deepened at single-cell and high-throughput levels (12). However, a major limitation of these techniques lies in their reliance on dissociated tissues or fixed tissue sections, which only provide static or post-mortem informations, failing to capture dynamic or live-cell data *in vivo*. Therefore, intravital imaging of tumor-TME interactions is essential. Moreover, the interactions between tumor cells and TME cells are increasingly recognized as potential therapeutic targets for effective cancer treatment (13).

Intravital imaging technologies enable real-time, longitudinal observation of interactions between tumor cells and other cells within the TME *in vivo*. Current techniques capable of achieving single-cell resolution *in vivo* include fluorescence- or bioluminescence-based optical imaging (14), magnetic resonance imaging (MRI) (15), photoacoustic imaging (16), positron emission tomography (PET) (17), and optical coherence tomography (OCT) (18). These technologies utilize fluorescent probes/proteins, radiotracers, or contrast agents to visualize and track specifically labeled cells or molecules at varying tissue depths and spatiotemporal resolutions. Among these, bioluminescence imaging, MRI, photoacoustic imaging, and PET typically offer spatial resolutions ranging from tens of micrometers to millimeters under conventional experimental conditions, which limits their ability to study cellular interactions at the single-cell level. OCT is suitable for superficial tissues. In contrast, IVM, combined with versatile imaging windows and diverse cell-labeling strategies, provides superior spatial resolution (approximately 1 submicron) and temporal resolution (sub-second), making it the preferred technique for researchers exploring cell-to-cell interactions in tumor initiation, progression, anti-tumor immunity, immune evasion, and adoptive cell therapy (19–21). Particularly for studying interactions between highly migratory immune cells and tumor cells, as well as cellular interactions during tumor cell invasion and metastasis, IVM enables dynamic, *in situ*, single-cell-level investigation of these processes (22, 23). This review summarises recent advances in IVM for uncovering the dynamic cellular interplay within the TME and its implications for advancing tumor biological research.

## 2 Advances in intravital microscopy imaging technology

IVM offers significant advantages in high spatial and temporal resolution, making it particularly suitable for live imaging at the single-cell level (24). In recent years, advancements in imaging technology, intravital imaging windows, cell labeling, and label-free imaging have enabled IVM to provide real-time, longitudinal three-dimensional (3D) cellular images, capturing data on morphology, motility, migration, and cell-cell interactions (25). Despite limitations such as restricted penetration depth, varying degrees of phototoxicity, and short observation durations, IVM delivers high-resolution spatiotemporal dynamic information that is critical for elucidating complex multicellular interactions within the TME. Consequently, the evolution of IVM technology has significantly enhanced our understanding of cellular interactions in the TME.

### 2.1 Different imaging microscopes

**Figure 1:**
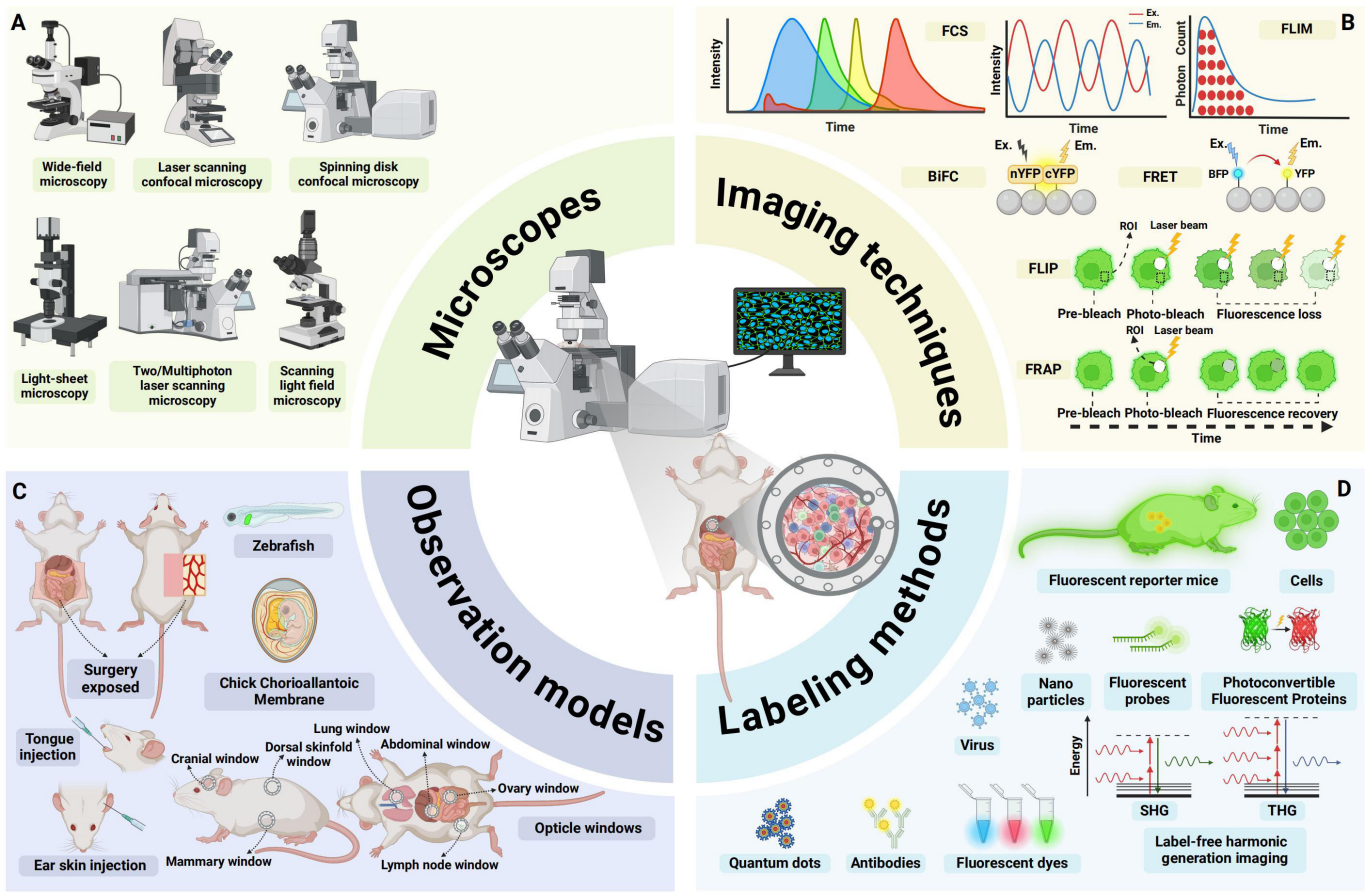
An overview of advances in intravital microscopy imaging technology. **(A)** Intravital imaging microscopes. **(B)** Specialized imaging techniques. **(C)** Appropriate observational models. **(D)** Diverse labeling methods. FCS, fluorescence correlation spectroscopy; FLIM, fluorescence lifetime imaging microscopy; BiFC, Bimolecular fluorescence complementation; FRET, Förster resonance energy transfer; FLIP, fluorescence loss in photobleaching; FRAP, fluorescence recovery after photobleaching; EX., excitation; EM., emission; ROI:region of interest; YFP:yellow fluorescent protein; BFP:blue fluorescent protein; SHG, second harmonic generation; THG, third harmonic generation; (Created with BioRender.com).

IVM encompasses a variety of modalities, including wide-field microscopy, confocal laser-scanning microscopy (CLSM), spinning-disc confocal microscopy (SDCM), Two-photon and multiphoton microscopy (2P/MPM), light sheet fluorescence microscopy (LSFM), and light field microscopy (LFM) (26) (**Figure 1A**). Each modality operates on distinct imaging principles, differing in light sources, illumination methods, imaging depth, effective field of view, photobleaching, and phototoxicity. For transparent or semi-transparent tissues, traditional wide-field fluorescence microscopy can be employed, though image quality is often compromised by out-of-focus light scattering. In contrast, modern LSFM utilizes two orthogonally arranged light sources to generate optical sections at the focal plane, yielding high-quality images (27). This also applies for transparent organisms like zebrafish xenografts and zebrafish cancer models, for which LSFM has been established as method of choice to image fast and gently (28). For most opaque tissues *in vivo*, confocal and two-photon microscopy are more suitable. Confocal microscopy employs a pinhole before the detector to block out-of-focus light, while 2P/MPM uses ultrafast pulsed lasers to restrict signal generation to the focal plane, thereby confining the signal to this plane (20). Both confocal and two-photon microscopy achieve high-resolution imaging in opaque tissues, albeit through different mechanisms. SDCM further enhances imaging speed by enabling simultaneous multi-point scanning. Additionally, 2P/MPM utilize low-energy infrared light, facilitating deep-tissue imaging (29, 30). Both 2P/MPM and LSFM mitigate photobleaching and phototoxicity, reducing cellular damage. However, despite suppressing background fluorescence via confocal detection (SDCM) and nonlinear excitation (2P/MPM), these methods still suffer from slow 3D imaging, residual phototoxicity (especially versus LSFM), and tissue aberration-induced resolution loss (30, 31). LFM addresses these issues by incorporating a pinhole or microlens array at the intermediate image plane to simultaneously record light intensity and angular information, enabling computational reconstruction of 3D structures. Dai et al. (32) used a scanning light field microscope that employs simultaneous 3D volume excitation and acquisition, significantly reducing unnecessary laser exposure and maximizing photon efficiency. This innovation drastically lowers phototoxicity and provides a novel approach for observing multicellular and multi-organelle interactions *in vivo*.

### 2.2 Specialized imaging techniques

To meet diverse experimental observation needs, a range of specialized imaging techniques has been developed (**Figure 1B**). For instance, Förster resonance energy transfer (FRET) leverages the energy transfer principle, which is highly dependent on the distance between two fluorophores, to study molecular interactions and spatial changes in macromolecules (33). Bimolecular fluorescence complementation (BiFC) involves splitting a fluorescent protein into two fragments and fusing them with target proteins to visualize protein-protein interactions (34). Fluorescence loss in photobleaching (FLIP) employs continuous photobleaching in a specific region, causing fluorescent molecules in surrounding areas to gradually lose fluorescence as they diffuse into the bleached zone. Fluorescence recovery after photobleaching (FRAP) involves photobleaching fluorescent molecules in a defined region and then monitoring the recovery of fluorescence, providing insights into protein dynamics and diffusion properties (35). Fluorescence correlation spectroscopy (FCS) analyzes fluctuations in fluorescence intensity within a small region, offering information on the concentration and dynamic interactions of fluorescent molecules (36). Fluorescence lifetime imaging microscopy (FLIM) measures the time fluorophores or phosphors remain in an excited state before emitting photons and returning to the ground state. Since the lifetimes of many fluorophores or phosphors are sensitive to environmental conditions such as temperature, pH, and oxygenation, FLIM can be used to monitor cellular metabolism and protein interactions (37, 38). These specialized imaging techniques facilitate a deeper understanding of intracellular and intercellular molecular dynamics and interactions.

### 2.3 Observational models

Due to tissue opacity and limited imaging depth of IVM, tissues and organs that cannot be directly observed often require surgical exposure or the establishment of imaging windows. For example, imaging windows can be created in the cranium (39), lungs (40), mammary fat pads (41), abdomen (42, 43), dorsal skinfold, or bone marrow (44) to obtain high-resolution images (45). Alternatively, transparent model organisms such as the chicken chorioallantoic membrane (CAM) (46), zebrafish (47), or easily accessible superficial sites like the tongue (48), ear skin (49), or skin flaps (50) can be utilized, depending on the specific experimental design (**Figure 1C**). Appropriate model systems are critical for IVM studies, which directly determines the biological relevance and experimental feasibility of *in vivo* imaging. **Table 1** provides a comparative analysis of the distinctive features across various observational models.

**Table 1:** Features across various observational models.

Model system	Transparency	Sample number	Whole organism observation	Subcellular imaging	Physiological comparison to humans	Reference
Surgical Exposure	Low	Low	No	Yes	High	(18)
Optical Window	Medium	Medium	Partial	Yes	High	(39–44)
Zebrafish	High	High	Yes	Yes	Moderate	(47)
Chicken Chorioallantoic Membrane	Medium-High	High	No	Limited	Low	(46)
Superficial Tissue Site	Medium	Medium	No	Yes	High	(48–50)

### 2.4 Diverse labeling methods

Combining a variety of *in vivo* cell and molecular labeling techniques can further enhance intravital imaging (**Figure 1D**). These include fluorescent proteins, dyes, antibodies, probes, nanoparticles, viruses, quantum dots, and specialized photoconvertible fluorescent proteins (PCFPs) such as Dendra2 (51) or Kaede (52), as well as genetically engineered mice with fluorescent markers. These labeling strategies can be combined to better visualize and distinguish target cells and molecules during imaging (53, 54). In addition to labeled imaging of cells or molecules *in vivo*, label-free imaging techniques can also be employed by leveraging the structural or molecular properties of tissues. Second harmonic generation (SHG) and third harmonic generation (THG) microscopy leverage nonlinear optical signals from biological tissues for label-free imaging (55). SHG visualizes non-centrosymmetric structures like collagen, myosin, and cell membranes, while THG detects interfaces and lipid-rich structures, enabling high-resolution, 3D imaging of tissue architecture without exogenous labels (56, 57).

## 3 Interactions between tumor cells

### 3.1 Shedding, aggregation and fusion between tumor cells

**Figure 2:**
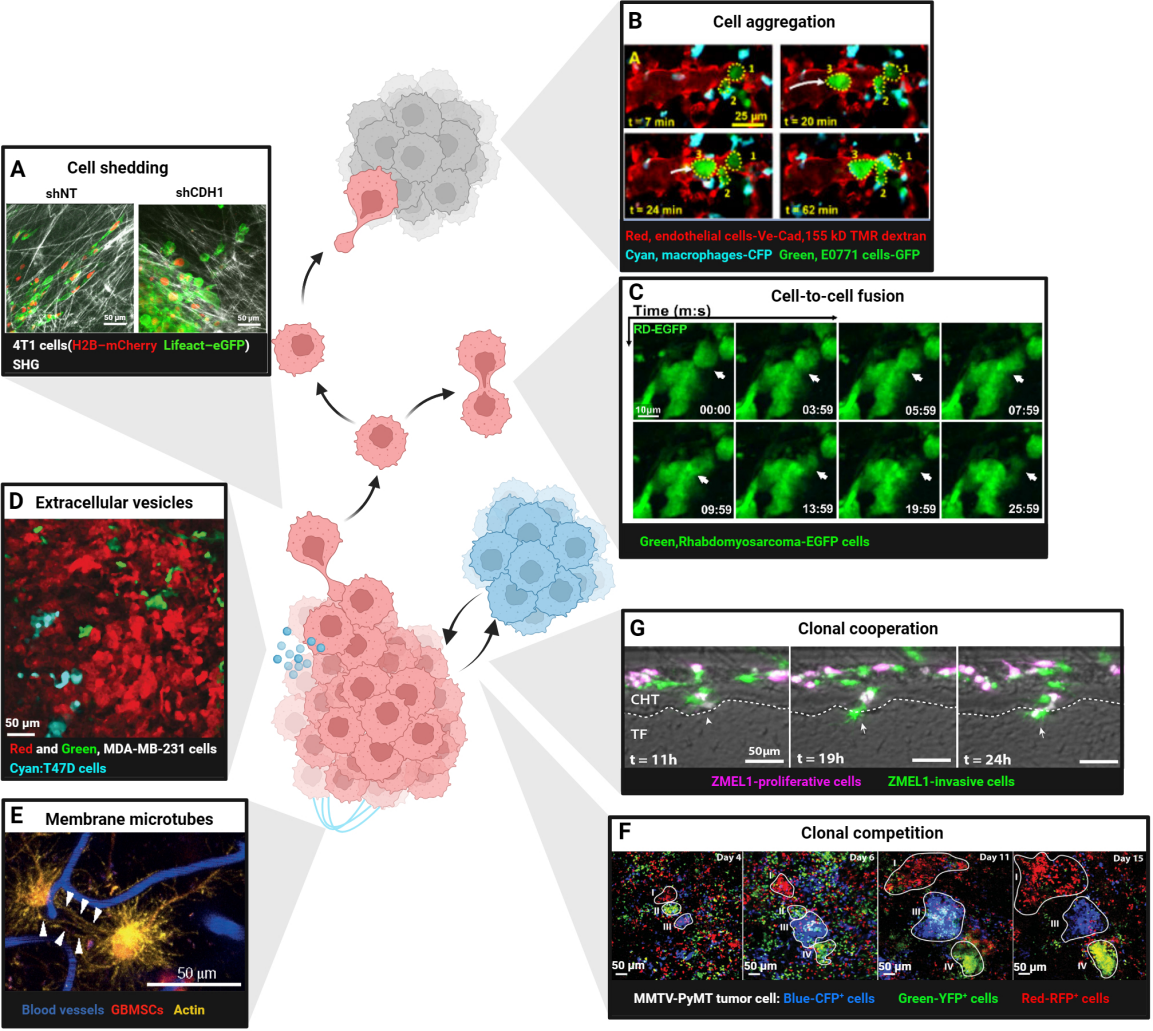
Intravital imaging in cellular interactions between tumor cells. **(A)** Shedding of single 4T1 cell expressing H2B–mCherry and Lifeact–eGFP from control (shNT) or E-cadherin knockdown (shCDH1) tissue subregions. Scale bars, 50 μm. Reproduced from Ilina et al. Adapted with permission from (41), copyright 2020 Springer Nature. **(B)** Aggregation of E0771-GFP breast cancer cells (numbers 1, 2 and 3; dotted yellow outlines) in the lung vasculature after intravenous injection. At t = 7 min, two tumor cells residing in the vasculature. At 20 min, a third tumor cell arrives. By t =62 min, the three cells form a cluster in the vasculature. Scale bars, 25 μm. TMR, tetramethylrhodamine; CFP, cyan fluorescent protein; GFP, green fluorescent protein. Reproduced from Entenberg et al. Adapted with permission from (40), copyright 2018 Springer Nature. **(C)** Fusion of rhabdomyosarcoma-EGFP cells in the tongue orthotopic xenografts. Scale bars, 10 μm. Reprinted from Hammoudeh et al. Adapted with permission from [45], copyright 2024 Elsevier. **(D)** Cre-reporter-expressing MDA-MB-231 cells that took up Cre-containing extracellular vesicles (EVs) released by T47D cells (cyan) switched from DsRed(red, before EV-uptake) to GFP (Green, after EV-uptake). Scale bars, 50 μm. Reproduced from Lodillinsky et al. Adapted with permission from (71), copyright 2016 Taylor & Francis. **(E)** Glioblastoma cells (red) in the mouse brain communicate via tumor microtubes(long membrane protrusions-rich in actin,yellow) that interconnect single tumor cells (arrowheads). Blood vessels are shown in blue. Scale bars, 50 μm. Reprinted from Osswald et al. Adapted with permission from (72), copyright 2015 Springer Nature. **(F)** Competition in four Confetti-labelled (blue: CFP+ cells, green: YFP+ cells, red: RFP+ cells) mammary carcinoma clones (white outline, regions I-IV).Different clonal growth patterns were observed in a series of intravital images at the indicated time points. Scale bars, 50 μm. Reproduced from Zomer et al. Adapted with permission from (76), copyright 2013 Oxford University Press. **(G)** ZMEL1-INV cells tightly adhered together, surrounded by ZMEL1-PRO cells, forming spatially structured heterogeneous PRO-INV clusters that cooperated in melanoma metastasis. Scale bars, 50 μm. Reprinted from Campbell et al. Adapted with permission from (77), copyright 2019 Elsevier. (Created with BioRender.com).

Normal epithelial cells maintain tissue integrity through tight junctions, adherens junctions (AJs), gap junctions and desmosomes (58, 59). Tumor cells lose apical-basal polarity and down-regulate E-cadherin, triggering epithelial–mesenchymal transition (EMT) and acquiring invasive mesenchymal motility (60, 61). IVM in breast-cancer xenografts showed that TGF-β drives single-cell/strand migration, whereas collective migration occurs when TGF-β signaling is absent (44) E-cadherin-mediated AJs provide stable intercellular adhesion. However, tumor transformation in epithelial cells triggers actin cytoskeleton reorganization, resulting in the remodeling of stable linear AJs into dynamic punctate or radial AJs. This structural alteration disrupts E-cadherin-mediated adhesion and enhances tumor cell invasion and migration (62). The plasticity of cancer invasion and metastasis depends on the ability of cancer cells to switch between collective invasion and single-cell migration modes, controlled by cadherin-mediated cell-cell junctions. Intravital two-photon microscopy demonstrated that down-regulation of E-cadherin and p120-catenin in MCF-7 and 4T1 cells disrupts cohesive collective invasion; when the local ECM becomes loose, cells detach and shift to single-cell migration (41) (**Figure 2A**). The plasticity of invasion therefore depends on cadherin-mediated junction dynamics that allow rapid switching between collective and individual motility. Dynamic intravital imaging revealed that PaTu8902 pancreatic cancer cells at the invasive front switch to an amoeboid morphology with elevated Myosin II activity, enabling ROCK-dependent penetration of collagen matrices (63). These data underscore how cytoskeletal reorganization and loss of E-cadherin actively promote tumor dissemination.However, the Rosenblatt lab discovered that Kras^V12^ cells also shed apical epithelial determinants by basal cell extrusion as a fast way to change plasticity and directly drive cell invasion (64).

Beyond shedding, tumor cells can aggregate into circulating tumor cell (CTC) clusters that possess 20–100-fold higher metastatic potential than single CTCs (65). IVM of triple-negative breast cancer revealed that CTC clusters form through rapid “touch-and-go” interactions near blood vessels, independent of collective migration (66). CD44-mediated homophilic binding drives this aggregation by up-regulating p21-activated kinase 2 (PAK2) and focal adhesion kinase (FAK). CRISPR/Cas9 knockout of CD44 reduces aggregation and lung colonization (66). Similarly, high-resolution lung-window imaging captured single E0771 cells arriving in pulmonary vessels, aggregating within 62 min and subsequently extravasating into alveoli (40) (**Figure 2B**). Finally, two-photon microscopy of sublingual rhabdomyosarcoma xenografts showed that EGFP-labeled tumor cells fuse approximately 10 days post-injection, forming elongated, muscle-like syncytia (67) (**Figure 2C**).

### 3.2 Tumor cell communication via extracellular vesicles and microtubes

Tumor cells release extracellular vesicles (EVs) that transfer proteins, lipids, nucleic acids and even mitochondria to recipient cells, thereby enhancing migration, immune evasion and therapy resistance (68, 69). Intravital imaging revealed that low-malignant T47D cells acquire aggressive traits after uptake of EVs from high-malignant MDA-MB-231 cells (70, 71) (**Figure 2D**). Ultra-long membrane microtubes observed in astrocytomas create multicellular networks that facilitate invasion and proliferation (72) (**Figure 2E**). glioblastoma cells connected by such tubes express higher levels of the stemness marker nestin and display radiation resistance (73). At a smaller nanoscale, tumor cells also can form transient tunneling nanotubes composed of actin and tubulin, requiring super-resolution imaging for detailed study (74, 75). These findings indicate that both EVs and microtubes actively promote tumor progression.

### 3.3 Competition and cooperation among tumor clones

Intratumoral heterogeneity fosters cooperative interactions that accelerate metastasis. Time-lapse confetti imaging in breast tumors revealed dominant clones with continuous expansion alongside subclones that regress or expand after a delay (76) (**Figure 2F**). In melanoma, transcriptomic analyses identified co-existing proliferative (PRO) and invasive (INV) subpopulations. In zebrafish embryos, INV cells coalesced into cores surrounded by PRO cells, forming spatially organized PRO-INV clusters that cooperated during dissemination; INV cells further enhanced the metastatic potential of less-aggressive PRO cells (77) (**Figure 2G**). Such clonal cooperation therefore constitutes a pro-tumorigenic mechanism that sustains both invasion and proliferation. However, clonal competition can suppress the outgrowth of aggressive subclones. Intravital imaging has documented instances where initially expanding clones are outcompeted and ultimately eliminated by neighboring populations (76) (**Figure 2F**), suggesting that competitive dynamics may restrict tumor progression under specific microenvironmental conditions.

## 4 Interactions between normal epithelial and tumor-transformed cells

**Figure 3:**
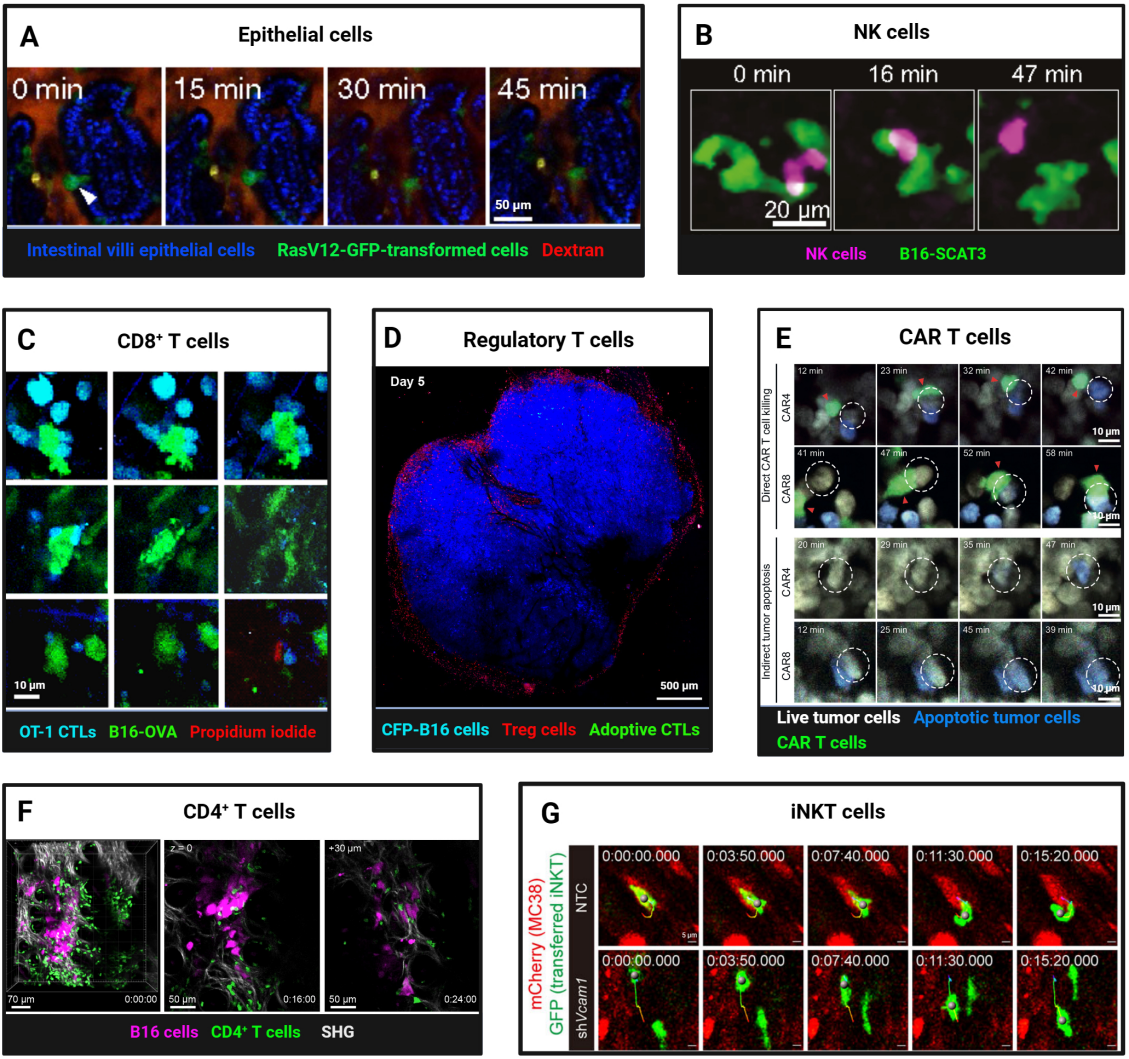
Intravital Imaging Reveals Dynamic Interactions of Normal Epithelial and Lymphoid Cells with Tumor Cells. **(A)** Two-photon imaging of apical extrusion of RasV12-expressing transformed cells from small intestine villi epithelial cells (arrowhead). Scale bars, 50 μm. Reproduced from Kon et al. Adapted with permission from (82), copyright 2017 Springer Nature. **(B)** Time-lapse intravital imaging captures NK cell contact and killing of B16-SCAT3 tumor cells in a lung metastasis mouse model. An NK cell and B16-SCAT3 cells are depicted in magenta and green, respectively. Scale bars, 20 μm. Reproduced and adapted with permission from (91), copyright 2022 Ichise et al. **(C)** Intravital imaging reveals *in vitro*-activated CFP^+^ OT-I CTLs killing of B16-OVA-GFP tumor cells. a Tumor cell death most often took the form of apoptosis, indicated by membrane blebbing (top). Less often lytic death occurred, as indicated by abrupt loss of cytoplasmic GFP and exposure of the nucleus (bottom). Rarely, both processes occurred simultaneously (middle row). Loss of membrane integrity is revealed by propidium iodide staining. Scale bars, 10 μm. Reproduced from Manaster et al. Adapted with permission from (98), copyright 2019 Springer Nature. **(D)** Foxp3-mRFP Tregs cells formed an “immunosuppressive ring” around the CFP-B16 tumor. Tregs cells and tumor cells are depicted in red and blue, respectively. Scale bars, 500 μm. Reproduced from and adapted with permission from (135), copyright 2016 Qi et al. **(E)** Representative two-photon time-lapse imaging captures CAR4/8 T cell-mediated direct and indirect tumor apoptosis. Red arrowheads mark CAR T cells engaged in cytotoxicity, white dashed circles denote apoptotic tumor cells killed by CAR T cells. Scale bars, 10 μm. Reproduced and adapted with permission from (107), copyright 2021 Boulch et al. **(F)** Two-photon intravital imaging confirmed that activated gDT-II CD4^+^ T cells inhibit B16.gD tumor cells in the absence of other lymphocytes. B16.gD tumor cells and gDT-II CD4^+^ T cells are depicted in magenta and green, respectively. Scale bars, 70 μm and 50 μm. Reprinted and adapted with permission from (111), copyright 2024 Bawden et al. **(G)** Time-lapse intravital images revealed the infiltration, intratumoral motility, and antigen scanning of GFP^+^ transferred iNKT cells in both Vcam1 knockdown and control (NTC) MC38-mCherry tumors. Scale bars, 5 μm. Reprinted and adapted with permission from (114), copyright 2024 Tian et al. (Created with BioRender.com).

In addition to immune surveillance, normal epithelial cells themselves constitute a frontline defence against malignant transformation through”epithelial defence against cancer”(EDAC) (78). Intravital imaging has captured this process across organs: in the pancreas, wild-type epithelia recognise Kras^G12D^-mutated neighbours by their elevated Ephrin type-A receptor 2(EPHA2) and, via down-regulation of the EPHA2-ephrin A pathway and E-cadherin, mechanically expel the transformed cells (79, 80). Inflammation and matrix stiffness modulate EDAC efficiency, thereby linking tissue context to tumour initiation and metastasis (81). loss of this mechanism permits survival and progression to pancreatic intraepithelial neoplasia. In the intestine, two-photon microscopy shows Ras^V12^ cells increase PDK4 to fuel their own apical extrusion (82) (**Figure 3A**), and caloric restriction augments this competition to further purge mutated clones (83). In adult mouse skin, sustained oncogenic β-catenin or Hras^G12V^ in hair-follicle stem cells produces exophytic outgrowths that are encircled and compressed by normal epithelia, leading to apoptosis and regression or re-differentiation into benign appendages (84).

## 5 Intravital imaging uncovers dynamic immune-tumor cell interactions

The tumor microenvironment (TME) exhibits dual immune regulation, where anti-tumor responses and immunosuppressive mechanisms coexist dynamically. IVM provides unprecedented spatiotemporal resolution to dissect these opposing processes (85, 86).

### 5.1 Anti-tumorigenic observations

#### 5.1.1 Natural killer cells

Natural killer (NK) cells exhibit highly dynamic behavior in tumors expressing the Natural Killer Group 2D (NKG2D) ligand Rae-1b. Brief interactions (270 seconds) between NK cells and tumor cells are sufficient to induce cytotoxicity, contrasting with the stable contacts (30 minutes) required for cytotoxic T lymphocytes. This rapid engagement and disengagement may facilitate NK cell dissemination within tumors and enable continuous tumor cell killing (87, 88). Using a FRET-caspase-3 apoptosis sensor in a live zebrafish model, Yang et al. (89) observed that NK cells can activate caspase-3 within 5–10 minutes of contact with tumor cells, leading to rapid tumor cell death within 40 minutes. Tumor-targeting antibodies can slow NK cell movement via Fc receptor binding, enhancing contact stability and improving cytotoxic efficacy (90). However, the killing effect of NK cells is not sustained. Combining ultrasensitive bioluminescence imaging with FRET-based two-photon microscopy, Ichise et al. (91) quantitatively analyzed the interaction between NK cells and tumor cells in a lung metastasis model of early-stage melanoma. Within four hours of reaching the lung capillary bed, approximately 50% of disseminated tumor cells (DTC) in contact with NK cells were eliminated (**Figure 3B**). NK cells rapidly activated extracellular signal-regulated kinase (ERK) and induced calcium influx upon contact with melanoma cells, leading to tumor cell death. All tumor cells were cleared by patrolling NK cells within 24 hours. However, after 24 hours, even upon contact, tumor cells were no longer killed. This was partly attributed to a decline in NK cell ERK activity from 68% to 8%, coupled with tumor cell downregulation of activating ligands such as CD155, enabling immune evasion and survival adaptation.

#### 5.1.2 CD8^+^ T lymphocytes

Activated CD8+ cytotoxic T lymphocytes (CTLs) recognize tumor antigens and release cytotoxic granules, thereby killing tumor cells. However, in most tumors, infiltrating CTLs express inhibitory receptors (PD-1, Tim-3, and Lag-3), which, upon binding to their ligands, suppress immune responses. In fact, many tumor cells exploit this immunosuppressive mechanism by expressing various ligands (PD-L1, PD-L2) that help them evade T cell attacks. Intravital imaging has revealed unique behaviors of CD8+ T cells during tumor immune surveillance and killing (92). The prerequisite for effective tumor cell recognition and killing by CTLs is the expression of cognate antigens by tumor cells. In the peripheral regions of tumors, activated CTLs migrate randomly at high instantaneous speeds, forming specific and stable contacts with tumor cells expressing cognate antigens, enabling antigen-specific recognition and immune killing. If tumor cells do not express cognate antigens, CTL infiltration is limited to the peripheral regions of the tumor, and they fail to maintain active migration, neither stopping nor forming stable contacts with tumor cells (93, 94).

CTL interactions with tumor cells can be categorized into three modes: stable interactions involving prolonged (>30 min) tight contacts (average speed < 2 µm/min), confined interactions characterized by slow migration around tumor cells (average speed: 2-3 µm/min), and serial interactions involving brief (<5 min) or no contact with tumor cells (average speed > 3 µm/min) (88, 92). Approximately 33.6% of CTLs exhibit stable interactions, while 18.1% exhibit confined interactions. These two subsets of CTLs are effective in recognizing and killing tumor cells. The remaining 48.3% of CTLs exhibit serial interactions (95, 96).

Using IVM to dynamically monitor caspase-3 and calcium ion sensor signals via FRET, we can observe the killing process of tumor cells by activated CTLs in real time. Although CTLs establish specific physical contact with tumor cells expressing cognate antigens, triggering apoptosis, this process can take several hours, indicating that the rate of tumor cell killing by CTLs in vivo is relatively slow. This is influenced by CTL functional heterogeneity, affinity differences, and immunosuppressive factors (T-regs, MDSCs, TAMs) (97, 98) (**Figure 3C**). Additionally, Breart et al. (99) found that it takes an average of 6 hours for one CTL to kill one tumor cell. In the immunotolerant liver, multiple CTLs are required to induce apoptosis in a single tumor cell, with each CTL killing an average of 1.24 ± 0.11 tumor cells per day, indicating limited killing efficiency in the liver. Calcium influx in tumor cells following CTL contact is an early response to apoptosis, occurring tens of minutes before caspase-3 activation(43). Compared to high-affinity T cells, low-affinity T cells have shorter contact times with tumor cells and dendritic cells. However, pretreatment with IL-12 enables low-affinity T cells to form stable contacts with tumor cells, enhancing antitumor effects (100). Moreover, intravital imaging of the bone marrow revealed that most interactions between CTLs and B-cell lymphoma are either ineffective or sublethal, highlighting tumor cell resistance to cytotoxic attacks and the heterogeneity of T cell activity (101). Additionally, IVM showed that CXCR6 expression enhances CTL survival and local expansion in the TME (102). CD44 is crucial for T cell navigation within the tumor stroma. Through its intracellular domain, CD44 recruits ezrin, radixin, and moesin (ERM) proteins to the rear of the cell, maintaining T cell polarity during migration without affecting interactions with target cells. CD44-deficient CTLs exhibit reduced migration rates in tumors and impaired ability to resume migration after contact with tumor cells, leading to decreased screening efficiency and potentially compromising tumor cell clearance (103). Beyond direct recognition and killing, intravital imaging experiments have shown that CD8+ T cells can modulate the behavior of distant tumor cells through IFNγ, including the generation of antigen-loss variants, increased PD-L1 expression, and tumor growth suppression. A small fraction of tumor cells recognized by T cells can trigger widespread IFNγ sensing, with bystander tumor cells also exhibiting significant expression of IFNγ-induced Katushka fluorescent protein, indicating that IFNγ signals can propagate over distances exceeding 800 µm within tumor tissue (104). Additionally, CTLs can limit tumor clonality and genetic diversity through epitope spreading. In female recipient mice, immune responses were observed not only against tumor cells with Y chromosomes but also against subdominant or cryptic epitopes, suggesting broader tumor antigen recognition facilitated by immune responses (93). Building on CTL-mediated antitumor immunity, strategies such as immune checkpoint inhibitors (93) blockade of immunosuppressive cytokines (43), laser immunotherapy (95), and modified Ankara vaccinia virus encoding scIL-12 (105) have been shown to enhance CTL infiltration and tumor cell killing. In a skin melanoma mouse model, tumor-specific epidermal CD69+ CD103+ tissue-resident memory T cells (TRMs) dynamically interacted with tumor cells, highlighting their role in immune surveillance. Depletion of TRMs led to tumor regrowth in approximately 20% of mice with latent melanoma, underscoring the importance of TRMs in maintaining tumor-immune equilibrium (106).

#### 5.1.3 CAR T cells

Chimeric antigen receptor (CAR) T cell therapy relies on the cytotoxic activity of tumor antigen-specific effector cells. Boulch et al. (107) observed in a B-cell lymphoma model that 72% of tumor apoptosis events in CAR8 T cell-treated mice were mediated by direct cell contact, whereas over 80% of apoptosis events in CAR4 T cell-treated mice occurred without direct interaction, suggesting that CD4+ CAR T cells are more effective in host immune activation but less so in direct tumor killing (**Figure 3E**). Further studies revealed that IFNγ release, rather than perforin, is the primary mechanism of tumor killing by anti-CD19 CD4+ CAR T cells (108). Live imaging showed that CAR T cells in mouse bone marrow exhibit rapid killing kinetics, completing tumor cell recognition, killing, and detachment within about 25 minutes. However, not all contacts trigger calcium signaling and killing, highlighting functional heterogeneity. Mathematical modeling confirmed that direct cytotoxicity is sufficient for rapid tumor clearance, emphasizing its importance in CAR T cell therapy (44). Radiotherapy and immune checkpoint inhibitors enhance CAR T cell-tumor cell interactions, improving antitumor efficacy. Whole-body irradiation (WBI) promotes rapid CAR T cell extravasation and local expansion, sustaining immune responses for up to 5 days (109). Anti-CTLA-4 and anti-PD-L1 therapies increase CD8+ T cell infiltration and CD44 expression, enhancing tumor cell contact and killing (100).

#### 5.1.4 CD4^+^ T cells

Unlike CD8+ T cells, CD4+ T cells were traditionally thought to mediate antitumor effects indirectly by regulating other immune cells (110). Recent studies, however, have demonstrated their direct antitumor activity. In a melanoma model, TCR-transgenic CD4+ T cells (gDT-II) infiltrated tumors and interacted with MHC II+ antigen-presenting cells carrying tumor debris, suppressing or eradicating tumors independently of other lymphocytes. Intravital imaging revealed that CD4+ T cells use TNF-α and FasL, rather than perforin, for cytotoxicity, with IFNγ playing a crucial protective role (111) (**Figure 3F**).

#### 5.1.5 Invariant natural killer T cells

Invariant Natural Killer T (iNKT) cells, which express both NK and T cell receptors, bridge innate and adaptive immunity, exhibiting potent antitumor activity. They kill tumor cells directly, activate other immune cells, and rejuvenate exhausted immune cells in the TME (112). Intravital imaging in a colorectal cancer liver metastasis model showed that α-galactosylceramide-activated iNKT cells significantly inhibited tumor growth, increased cell numbers and granularity, and enhanced tumor cell contact. However, tumor cells eventually evade immune surveillance, reducing therapeutic efficacy over time (113). Blocking VCAM1-CD49d interactions enhances iNKT cell infiltration and motility, improving antigen scanning and antitumor immunity (114) (**Figure 3G**).

#### 5.1.6 Tumor-associated macrophages

**Figure 4:**
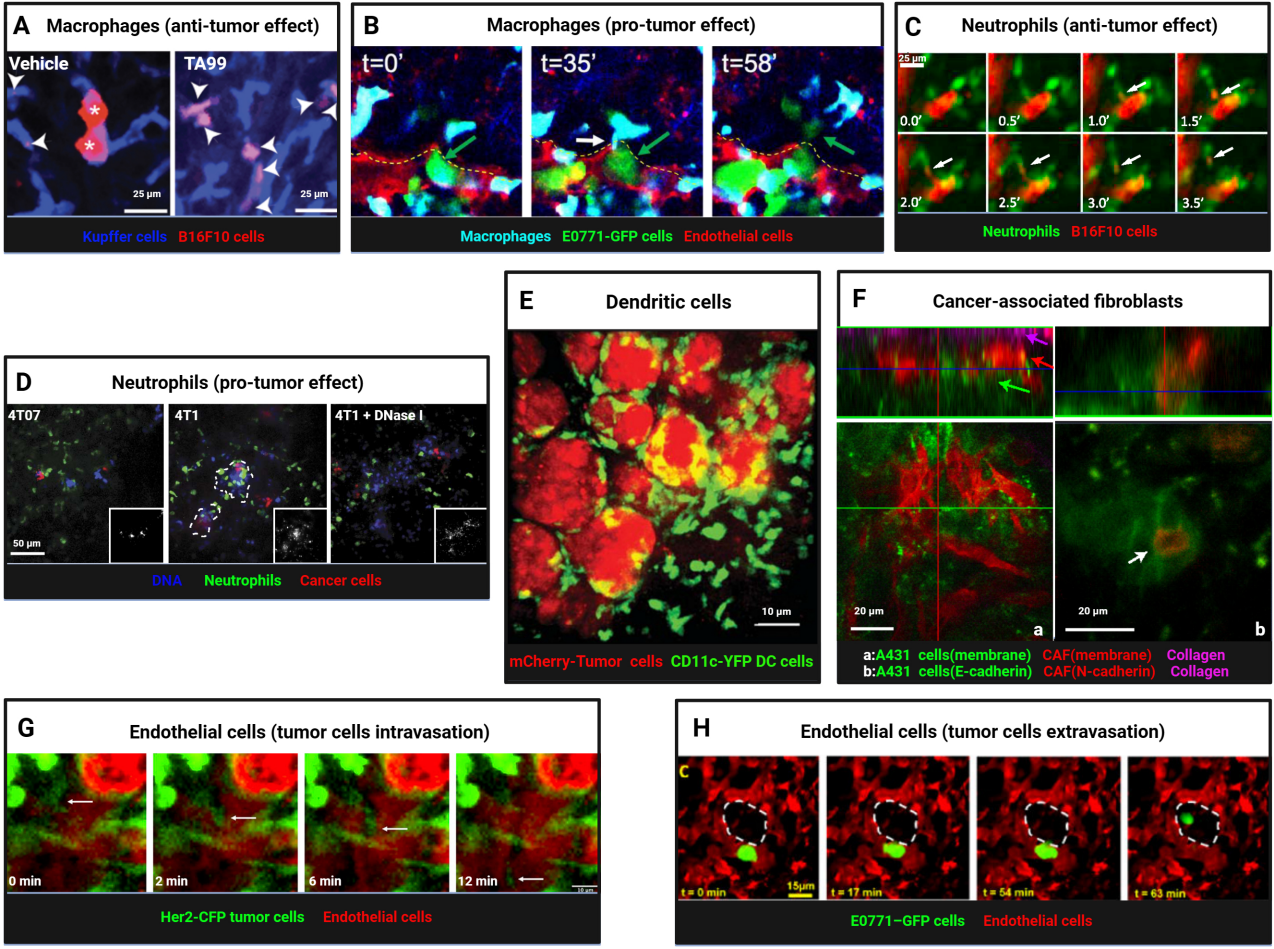
Intravital imaging reveals dynamic interactions between myeloid cells, stromal cells, and tumor cells. **(A)** Kupffer cells (blue) and B16F10 tumor cells (red) were visualized in livers of vehicle- or TA99-treated mice, revealing tumor cell particles engulfed by Kupffer cells (arrowheads) and intact tumor cells (asterisks). Scale bars, 25 μm. Reproduced and adapted with permission from (117), copyright 2014 Gül et al. **(B)** Time-lapse imaging captured macrophages (blue) interacting with E0771-GFP tumor cells (green) during extravasation, showing thin membranous connections (TMC, white arrow) and subsequent tumor cell extravasation into lung parenchyma. A yellow dashed line marks intact endothelium (red). Reprinted and adapted with permission from (140), copyright 2023 Genna et al. **(C)** Neutrophils (green) engaged in trogocytosis with TA99-opsonized B16F10 cells (red), with arrows indicating membrane fragment uptake. Scale bars, 25 μm. Reprinted and adapted with permission from (128), copyright 2018 Matlung et al. **(D)** Confocal imaging revealed enhanced neutrophil infiltration (green) in metastatic mCherry-4T1 tumors (red) compared to nonmetastatic 4T07 tumors (red), with NET-like DNA structures (blue), sensitive to DNase I, prominent around 4T1 cells. Scale bars, 50 μm. Reproduced and adapted with permission from (145), copyright 2016 Park et al. **(E)** Imaging of mCherry+ CD11c-YFP+ dendritic cells taking up tumor antigens (red-mCherry). Scale bars, 10 μm. Reprinted from Engelhardt et al. Adapted with permission from (131), copyright 2012 Elsevier. **(F)** Imaging of interactions between CAF and tumor cells. Tumors growing in mouse ears, with **(a)** A431 cells (green), CAFs (red), and collagen second harmonic signal (magenta). Arrows mark different tumor components. **(b)** A431-E-cad-Ruby cells (green) and vulval CAF-N-cad-GFP cells (red). A white arrow marks cell-cell contact. Scale bars, 20 μm. Reprinted from Labernadie et al. Adapted with permission from (159), copyright 2017 Springer Nature. **(G)** Time-lapse imaging captured Her2-CFP tumor cells (green) intravasating into blood vessels (red, TRITC dextran, arrow). Scale bars, 10 μm. Reproduced from Di Martino et al. Adapted with permission from (165), copyright 2019 PORTLAND PRESS. **(H)** A lung window model enabled visualization of tumor cell extravasation in spontaneous metastasis, with tumor cells crossing the endothelium into alveolar spaces (dashed white outline). Scale bars, 15 μm.Reprinted from Entenberg et al. Adapted with permission from (40), copyright 2018 Springer Nature. (Created with BioRender.com).

Tumor-associated macrophages (TAMs) are the most abundant immune cells in the TME, constituting up to half of the immune cell population (115). TAMs exhibit remarkable phenotypic and functional plasticity, recruited to tumor regions by various stimuli (chemokines, cytokines, exosomes) and polarized into M1 type (pro-inflammatory and anti-tumor) and M2 type (anti-inflammatory and pro-tumor) (116) The anti-tumor function of TAMs is primarily mediated through direct phagocytosis of tumor cells upon contact. Gül et al. (117) used IVM to observe that macrophage-mediated phagocytosis of tumor cells depends on the opsonization by specific monoclonal antibodies and the presence of FcγRI (high-affinity IgG-binding Fc receptor) and FcγRIV (low-affinity IgG-binding Fc receptor). In C57BL/6 mice inoculated with syngeneic B16F10 tumor cells, treatment with the TA99 monoclonal antibody (anti-gp75) enabled Kupffer cells (KCs) in the liver to rapidly recognize and phagocytose tumor cells, degrading them into small particles. In contrast, control-treated mice showed Kupffer cells in contact with tumor cells but unable to phagocytose them, allowing tumor cells to proliferate and form large clusters (**Figure 4A**). Similarly, in a CD20 monoclonal antibody-treated B-cell malignancy mouse model, Montalvao et al. (118) confirmed that KCs mediate the abrupt arrest and subsequent phagocytosis of B cells in the hepatic sinusoids. Beyond Kupffer cells in the liver, IVM has also observed microglia in the central nervous system (39) and macrophages in metastatic sites phagocytosing tumor cells (119). The phagocytic capacity of TAMs is not static. Liu et al. (120) observed in a late-stage liver metastasis model that KCs were significantly reduced in the tumor core and periphery, forming “KC dark zones,” which impaired their ability to clear tumor cells. CRISPR/Cas-mediated re-editing of KCs to proliferate and re-enter tumor tissue enabled effective tumor cell elimination through nibbling, reshaping the tumor immune microenvironment. In a mouse model of lung cancer brain metastasis, Zhang et al. (39) used two-photon microscopy through a cranial window to observe that TAMs/microglia phagocytosed escaping tumor cells in the early stages, but this effect diminished in later stages of metastasis formation. IVM has also revealed TAMs taking up exosomes secreted by tumor cells (121), phagocytosing particles released by tumor cells (122, 123), and engulfing collagen fibers in the tumor stroma (124). Dalla et al. (125) found in a breast cancer lung metastasis model that DTCs in early-stage tumors (HER2+ DTCs) predominantly maintained prolonged contact (70%) with alveolar macrophages, while late-stage tumors (E0771 DTCs) showed more transient interactions. Alveolar macrophages, upon contact with DTCs, induced dormancy via the TGF-β2/TGF-βRIII signaling pathway. Depletion of alveolar macrophages or downregulation of TGF-β2 receptors triggered metastatic awakening.

#### 5.1.7 Neutrophils

Neutrophils recruited by tumor-secreted chemokines exhibit high functional plasticity (126). In anti-tumor roles, zebrafish models revealed that neutrophils are recruited by Hras^G12V^-transformed tumor cells via hydrogen peroxide signaling, forming cytoplasmic traction fibers and phagocytosing transformed cells (127). Matlung et al. (128) used SDCM to observe neutrophils directly phagocytosing TA99-opsonized B16F10 tumor cells (trogocytosis), a process enhanced by blocking CD47-SIRPα interactions (**Figure 4C**). Stimulation with microbial agents significantly increased neutrophil expansion, migration speed, and displacement, enhancing their dynamic migration and clustering around tumor cells and further boosting anti-tumor effects (129).

#### 5.1.8 Dendritic cells

Dendritic cells (DCs) are specialized antigen-presenting cells within the tumor microenvironment, primarily responsible for capturing, processing, and presenting antigens. They express co-stimulatory molecules and activate T cells, thereby initiating immune responses (130). Engelhardt et al. (131) crossed polyoma middle T antigen (PyMT) ChOVA mice with mice expressing yellow fluorescent protein (YFP) under the control of the CD11c promoter. Through real-time intravital imaging, they observed that CD11c-YFP+ DCs within 5 mm of the tumor (proximal) were engaged in tumor antigen uptake, while those beyond 5 mm (distal) exhibited dynamic environmental sampling behaviors (**Figure 4E**). Ruhland et al. (132) used multiphoton microscopy to observe DCs in the tumor-draining lymph nodes of mice, noting that these cells form tight, synapse-like contacts to transfer tumor antigens. These contacts are dynamic and can persist for extended periods. Headley et al. (123) employed two-photon microscopy to study CTCs in the lungs of mice, discovering that CTCs produce and release micron-sized tumor microparticles (cytoplasts) that can move within or adhere to blood vessel walls. Conventional dendritic cells (cDCs) interact with CTCs, providing anti-metastatic protection. Additionally, intravital imaging experiments revealed that tumor cells secreting GRP78 (sGRP78) interact with DCs and macrophages in the liver, inhibiting DC activation and inducing macrophage polarization toward the M2 phenotype, ultimately promoting tumor immune tolerance (133).

### 5.2 Pro-tumorigenic observations

#### 5.2.1 Regulatory T cells

Regulatory T cells (Tregs) in tumor are associated with poor prognosis and immunotherapy resistance (134). *In vivo* imaging revealed that Tregs can form an “immunosuppressive ring” around solid tumors. Combination therapy with cyclophosphamide and cell therapy disrupts this ring, promoting antitumor immune responses by enhancing CTL accumulation, dendritic cell infiltration, and transient activation of endogenous tumor-infiltrating immune cells (135) (**Figure 3D**).

#### 5.2.2 Tumor-associated macrophages

IVM in primary breast cancer and lung metastasis sites revealed that tyrosine kinase with immunoglobulin-like and EGF-like domains 2 (Tie2) high macrophages and mammalian-enabled (MENA)-overexpressing tumor cells directly contact endothelial cells near blood vessels, forming specialized tumor microenvironment metastatic portals (TMEMs). Macrophages increase vascular permeability through the vascular endothelial growth factor (VEGF) A signaling pathway, facilitating tumor cell transendothelial migration and extravasation (136, 137). Near TMEMs, macrophages induce stem-like properties in non-stem tumor cells via the Notch-Jagged signaling pathway (138), activating programs related to tumor cell dormancy and invasion (139). Similar TMEM structures are also present in metastatic sites (40). Additionally, Genna et al. (140) discovered that extravascular macrophages and intravascular tumor cells establish direct connections through tunneling nanotube-like membrane connections (TMCs). Knockout of the M-Sec (TNFAIP2) gene impaired TMC formation, reducing macrophage-tumor cell interactions and decreasing distant metastasis, suggesting that such direct contacts are critical for tumor cell transendothelial migration and metastasis (**Figure 4B**). Knockout of the IL-4 receptor (IL4rα) reduced physical interactions between tumor cells and IL4rα-deficient macrophages, correspondingly decreasing metastatic foci, indicating that IL-4 regulates macrophage-tumor cell interactions (141).

#### 5.2.3 Neutrophils

Neutrophils can play dual roles in either anti-tumor or pro-tumor activities depending on the stage of tumor progression (126). Pathologically activated neutrophils, also known as polymorphonuclear myeloid-derived suppressor cells (PMN-MDSCs), are key components of the immunosuppressive microenvironment, strongly inhibiting lymphocyte-mediated cytotoxicity and promoting tumor progression through multiple mechanisms (142). Spicer et al. (143) used SDCM to observe CTCs adhering to neutrophils trapped in hepatic sinusoids, promoting lung cancer liver metastasis. This adhesion was mediated by Mac-1/ICAM-1, suggesting neutrophils act as bridges facilitating tumor cell interactions with liver parenchyma. Depleting neutrophils reduced early tumor cell adhesion in the liver. However, depletion of peripheral blood neutrophils using Gr-1 or Ly-6G antibodies left behind resistant and migratory immature Ly-6G+ cells in tumors (144), highlighting challenges in neutrophil-depletion-based therapies. Neutrophils release DNA fiber networks known as neutrophil extracellular traps (NETs), which capture tumor cells to support metastatic progression. Park et al. (145) found that metastatic 4T1 breast cancer cells induce NET formation in the lungs (**Figure 4D**). Kamioka et al. (146) used FRET technology to show that osteopontin (OPN) plays a key role in activating the ERK pathway in PMNs around 4T1 mammary tumor cells, inducing NETosis and promoting metastasis. NETosis inhibitors like DNase I (a NET-degrading enzyme) suppressed 4T1 lung metastasis, while strategies such as NEi (a NET inhibitor) or PAD4 gene knockout (a key enzyme for NET formation) significantly inhibited tumor cell metastasis in preclinical lung and colon cancer models (147). Additionally, Liane et al. (148) observed that neutrophil movement speeds in lung capillaries were higher than in melanoma (B16F10-mCherry) and breast cancer (E0771-iRFP720) lung metastases. In a mouse oropharyngeal cancer (MOPC) model, intratumoral neutrophil movement was slower than in the tumor periphery, regulated by the CXCR2 signaling pathway (149). Leveraging the tumor-homing ability of neutrophils, they hold promise as drug delivery vehicles, with delivery efficiency depending on nanoparticle type (150). Intravital imaging has significantly expanded our understanding of neutrophil-tumor cell interactions. Using antibodies against cell markers like Gr-1 and Ly6G or fluorescent reporter genes, neutrophils or PMN-MDSCs can be tracked *in vivo*. Although they share common origins and many morphological and phenotypic features, their roles in the tumor microenvironment often differ (151). Most recently, Egeblad’s lab reported a tumour-elicited Ly6G^High^Ly6C^Low^ neutrophil population that was unable to extravasate in response to inflammatory challenges can form intravascular NETs more efficiently and occlude tumour vessels, generate pleomorphic necrosis and induce perinecrotic EMT to promote metastasis, whereas genetic or pharmacological NET inhibition reduces both necrosis and metastatic spread (152).

### 5.3 Migratory behavior

We can also observe the migration behavior of immune cells using Intravital Microscopy. CTLs exhibit different behaviors in various regions of the tumor microenvironment as they migrate from the periphery to the core of the tumor tissue. In the collagen-rich peripheral regions, CTLs adopt an elongated, polarized morphology and migrate randomly along collagen fibers or blood vessels at high instantaneous speeds (4.27 ± 0.11 µm/min), displaying Lévy-like trajectories. Near the tumor cell regions, T cells reduce their speed, adopt a morphology with extended lamellipodia at the front and shortened uropods at the rear, and exhibit faster morphological changes, suggesting active probing of the local environment. In the core regions of the tumor, CTLs display a more compact morphology, dynamically forming and retracting pseudopods. Some T cells in close contact with tumor cells remain stationary and round, indicative of cell-cell interactions. After contact with tumor cells, CTLs in regions of tumor cell necrosis or apoptosis resume migration and polarized morphology (92, 96, 97, 153). IVM has also provided insights into the dynamics and influencing factors of CTL motility. In the 4T1 breast cancer model, tumor-infiltrating lymphocytes (TILs) exhibit gradually increasing motility, with higher motility inside the tumor than outside. In the MDA-MB-231 model, TIL motility initially increases before gradually declining. Significant differences in TIL motility between MDA-MB-231 and MCF-7 models suggest a correlation between PD-L1 expression levels and TIL motility (154). CTL motility also varies with proximity to blood vessels. In a melanoma mouse model, OT-I CTLs predominantly cluster around blood vessels, migrate rapidly, and form stable conjugates with B16-OVA tumor cells, effectively killing them in a contact- and perforin-dependent manner. In contrast, migration slows in hypoxic regions distant from blood vessels, indicating that oxygen supply is critical for CTL motility. Intravital imaging revealed that CTL movement halts immediately when blood flow is temporarily blocked and resumes rapidly upon flow restoration, underscoring the dependence of CTL migration on oxygen levels near blood vessels (98). Phosphorescence lifetime imaging microscopy (PLIM) also showed that hypoxia slows the motility of tumor-infiltrating T cells, while oxygen supplementation significantly enhances their motility (155).

## 6 Intravital imaging uncovers cancer-associated fibroblast-tumor cell interactions

Cancer-associated fibroblasts (CAFs) can infiltrate tumor tissues and promote tumor invasion and metastasis through various mechanisms (156). Fsp1+ fibroblasts exhibit distinct migratory properties in different regions of the tumor stroma, including the tumor margin and interior (157). Fibroblasts can promote breast cancer cells to adopt either single-cell/chain migration or collective migration phenotypes, depending on the TGF-β signaling status of epithelial cells. Using TPM, it was observed that tumors with TβRII knockout collectively migrate along blood vessels, suggesting that fibroblasts influence breast cancer cell invasion behavior via TGF-β signaling (46). Ferrari et al. (158) conducted intravital imaging and SHG imaging in CD-1 nude mouse models, demonstrating that DKK3 expression in CAFs activates Yes-associated protein (YAP) and transcriptional coactivator with PDZ-binding motif (TAZ) signaling, thereby enhancing tumor cell migration and invasion. Labernadie et al. (159) found that CAFs can exert mechanical forces through heterotypic adhesion with cancer cells, particularly via interactions between E-cadherin and N-cadherin, promoting collective cancer cell invasion and metastasis. Targeting this mechanical heterotypic adhesion offers a novel therapeutic strategy for combating cancer invasion and metastasis (**Figure 4F**). Furthermore, Vennin et al. (160) utilized a cyclin-dependent kinase (CDK) 1-FRET biosensor to observe interactions between TP53-mutant cancer cells and CAFs in live tumors, revealing that these interactions delay cancer cell responses to standard chemotherapy.

## 7 Intravital imaging uncovers endothelial-tumor cell interactions

Tumor vasculature plays a pivotal role in tumor dissemination, metastasis, and treatment, with the interaction between tumor cells and vascular endothelial cells being particularly crucial (161). Tumor cells that undergo EMT exhibit enhanced invasive and migratory capabilities, and their migration toward and intravasation into blood vessels are critical steps in tumor dissemination and metastasis. Tumor cells that have undergone EMT express high levels of the hepatocyte growth factor (HGF) receptor (cellular-mesenchymal epithelial transition factor(c-Met)) and tend to accumulate near blood vessels, where they can interact with the vessel wall through extended membrane structures. This interaction suggests that blood vessels play a significant role in tumor cell migration, particularly in promoting the motility and invasiveness of EMT-transformed cells (162). Leung et al. (163) observed that tumor cells align linearly and migrate directionally along a concentration gradient of HGF/c-Met signaling secreted by endothelial cells, moving toward TMEM sites via ECM fibers rich in fibronectin and collagen I. This process, termed “streaming migration” is driven by chemotactic signals. Tumor cells overexpressing Mena, pro-angiogenic TIE2^hi^/VEGF^hi^ macrophages, and vascular endothelial cells form tight associations at TMEM sites, facilitating the transendothelial migration of tumor cells. *In vivo* single-cell resolution imaging and photoconversion lineage tracing have revealed how individual tumor cells interact with endothelial cells and pericytes at TMEM sites, leading to transient increases in vascular permeability and subsequent intravasation into the bloodstream. IVM has captured the directed migration of tumor cells toward blood vessels and their transient intravasation events at TMEM sites (136, 137, 164, 165) (**Figure 4G**). Karagiannis et al. (166) demonstrated *in vivo* that chemotherapy can increase local vascular permeability at TMEM sites and promote tumor cell intravasation, a process critical for distant metastasis. Using translucent zebrafish embryos and high-resolution confocal microscopy, Stoletov et al. (167) observed that MDA-435 tumor cells expressing the RhoC gene form dynamic membrane protrusions and bleb-like structures, adopting a primitive amoeboid invasion mode. These cells accumulate at sites of vascular remodeling rather than intact vessels, secreting VEGF to induce the opening of remodeled vessels, which serve as entry points for RhoC-expressing tumor cells to penetrate the vessel wall and enter the circulation. These *in vivo* imaging studies highlight VEGF-induced vascular remodeling and the interaction between tumor cells and vascular openings during intravasation. Confocal optical sections and 3D reconstructions further illustrate the contact points between tumor cells and the endothelial layer, as well as the integration of tumor cell membranes with vascular openings. Additionally, endothelial expression of cellular communication network factor 1 (CCN1) promotes stable adhesion of tumor cells to blood vessels (168), while ephrin-B2 expression drives the perivascular invasion of tumor stem cells (169).

Extravasation of CTCs is another critical step in cancer metastasis. To better understand how tumor cells arrest, adhere to endothelial cells, and extravasate through the vessel wall, Stoletov et al. (170) used intravital imaging in translucent zebrafish embryos to observe the behavior of transplanted MDA tumor cells during vascular arrest and extravasation. They found that tumor cell extravasation is a highly dynamic process. Tumor cells rely on integrin subunit β 1 (ITGB1) to adhere to the vessel wall and arrest within the vasculature. A subset of arrested tumor cells actively migrates against the blood flow along the luminal surface of the endothelium before extravasating through narrow vascular constrictions into the extravascular matrix (24%, 16 out of 65 cells were migratory, with a maximum speed of 2.01 µm/min and an average migration speed of 1.26 ± 0.56 µm/min). Tumor cells induce vascular remodeling by altering endothelial cell alignment and junctions, a process that does not involve vascular leakage but is closely associated with metastatic potential. Expression of pro-metastatic genes such as Twist and VEGFA enhances the intravascular migration and extravasation capabilities of tumor cells. Silencing ITGB1 reduces VEGF-induced extravasation, indicating that this process is ITGB1-dependent. VEGFR inhibitors can reduce endothelial remodeling induced by shear stress, thereby decreasing CTCs extravasation and metastasis (171). Using the CAM model and IVM, Follain et al. (172) also observed the extravasation process of tumor cells. Tumor cells first form invadopodia enriched with specific proteins (membrane-type 1 matrix metalloproteinase (MT1-MMP) and Tyrosine Kinase Substrate with Five SH3 Domains (Tks5)), which penetrate the endothelial layer and invade the extravascular matrix. Inhibiting invadopodia formation and function significantly reduces tumor cell extravasation and metastasis. Entenberg et al. (40) used lung window imaging to capture continuous intravascular imaging of E0771 mammary tumor cells approximately 60 minutes after tail vein injection. They observed an invasive protrusion crossing the endothelium into the alveoli 16 minutes after imaging began, with the entire transendothelial migration process taking about 62 minutes. This process was also observed in spontaneous metastasis models, where tumor cells extravasated from blood vessels into alveolar spaces, forming micrometastases (**Figure 4H**). Additionally, endothelial expression of S1PR1 influences tumor vascular integrity, affecting angiogenesis, tumor growth, and hematogenous metastasis (173). Platelets can enhance tumor cell adhesion to the vessel wall, strengthening the interaction between tumor cells and endothelial cells (174), while high YAP expression promotes the active migration of CTCs retained in capillaries, allowing them to traverse capillary networks and re-enter the systemic circulation, thereby facilitating metastasis (175). During the process of extravasation from the endothelial barrier to the extravascular matrix and subsequent metastasis to distant organs, tumor cells undergo a physical transition from a more flexible to a more rigid state to adapt to the local microenvironment (176). Furthermore, VEGF-dependent release of thromboxane A2 (TXA2) by endothelial cells can trigger calcium transients in tumor cells, leading to prostaglandin E2 (PGE2) secretion and promoting tumor immune evasion (177).

These findings provide unprecedented high-resolution *in vivo* imaging data that deepen our understanding of tumor cell metastasis mechanisms and offer new insights into cancer treatment and therapeutic resistance. *In vivo* imaging experiments with oncolytic herpes simplex virus-1 (oHSV) revealed that tumor cells distant from blood vessels persistently express the virus, while those near blood vessels effectively clear it, suggesting that the vascular microenvironment may influence the efficacy of oncolytic viruses. Modulating tumor vascular activity could enhance the virus’s tumor-killing effects (178). Tumor progression is accompanied by changes in the tumor vascular network phenotype and the polarization state of perivascular macrophages. In early tumor growth, a highly functional vascular network with normal diameter and rich branching structures forms. As the tumor progresses, *in vivo* imaging shows that the vascular pattern evolves into dilated, leaky vessels with reduced branching complexity and impaired perfusion. Perivascular macrophages shift from a M1 phenotype to a VEGF-A-secreting M2 phenotype (179). Although high-dose radiation can induce apoptosis in tumor vascular endothelial cells, its effects on tumor vascular structure are largely limited to small, non-perfused vessels, with minimal impact on the perfused vascular network (180).

## 8 Summary and outlook

Tumors are complex systems, and every stage of tumorigenesis and progression involves intricate interactions among different cell types within this system. IVM is an advanced tool for decoding these interactions, helping to elucidate key steps in the complex interplay between cells and offering new perspectives and potential therapeutic targets for cancer treatment (181). Real-time observation of cell interactions in the live TME differs significantly from traditional ex vivo tissue or *in vitro* studies. This review focuses on the interactions between tumor cells and other cells in the TME, although certain cell types, such as B cells, remain understudied in terms of *in vivo* visualization.

It is important to acknowledge the limitations of current intravital imaging technologies, including limited spatial fields of view, insufficient imaging depth, and challenges in labeling and distinguishing specific cell types or functional subpopulations. These issues represent future challenges for intravital imaging. Moreover, the rapid development of single-cell multi-omics, spatial omics, and sequencing-based, proximity labeling, or synthetic circuit technologies allows for deeper exploration of the molecular mechanisms underlying cell-cell interactions and communication (182). Additionally, the advent and application of super-resolution imaging techniques, such as expansion microscopy (ExM), which physically enlarges specimens to bypass the optical diffraction limit, enables nanoscale visualization of subcellular structures and their spatial relationships. When combined with single-cell multi-omics or spatial transcriptomics, ExM may further bridge the gap between molecular profiling and ultrastructural context within the TME (183–185). Meanwhile, novel strategies like Tartrazine (186) to increase transparency in live animals, and smart imaging technologies will become increasingly available for event-based detections in cancer, and allow multi-scale imaging of a whole organism and sub-cellular regions simultaneously to study cancer across scales (187). The advancement and integration of these technologies will undoubtedly provide a more comprehensive understanding of the rules governing cell interactions during tumorigenesis and progression, offering further insights into the mechanisms of precision cancer therapy and potentially improving clinical outcomes.
